# ECG Dispersion Mapping in Preclinical Diagnosis of Cardiovascular Diseases

**DOI:** 10.17691/stm2020.12.5.10

**Published:** 2020-10-28

**Authors:** E.Yu. Esina, A.A. Zuikova, I.S. Dobrynina, V.V. Lyutov, V.N. Tsygan

**Affiliations:** Professor, Department of Polyclinic Therapy; Voronezh State Medical University named after N.N. Burdenko, 10 Studencheskaya St., Voronezh, 394036, Russia;; Professor, Head of the Department of Polyclinic Therapy; Voronezh State Medical University named after N.N. Burdenko, 10 Studencheskaya St., Voronezh, 394036, Russia;; Associate Professor, Department of Polyclinic Therapy; Voronezh State Medical University named after N.N. Burdenko, 10 Studencheskaya St., Voronezh, 394036, Russia;; Professor, Deputy Chief; Military Medical Academy named after S.M. Kirov, 6 Akademika Lebedeva St., Saint Petersburg, 194044, Russia; Professor, Head of the Department of Pathological Physiology Military Medical Academy named after S.M. Kirov, 6 Akademika Lebedeva St., Saint Petersburg, 194044, Russia

**Keywords:** ECG dispersion mapping, risk factors for cardiovascular diseases, somatoform dysfunction of the ANS

## Abstract

**The aim of the study** was to develop a method for diagnosing pre-nosological changes in the electrophysiological state of the myocardium in patients with somatoform dysfunction of the autonomic nervous system (SDANS) and risk factors for cardiovascular diseases using the ECG dispersion mapping method.

**Materials and Methods.:**

The study involved 109 male patients, 58 of them with SDANS, and 51 were healthy subjects. The patients with SDANS had the following risk factors for cardiovascular diseases, in decreasing order: stress (71% of cases), low physical activity (59%), smoking (57%), overweight and obesity (43%), anxiety (41%), low consumption of vegetables and fruit (36%), lack of extra aerobic physical activity (36%), excessive alcohol consumption (34%), depression (26%), total cholesterol ≥5 mmol/L (23%), and heart rate ≥80 (9% of the cases). All the subjects underwent clinical examination, laboratory investigation, ECG, ECG dispersion mapping, heart rate variability monitoring.

**Results.:**

Using the method of ECG dispersion mapping allowed a way for diagnosing pre-nosological changes in the electrophysiological state of the myocardium in male patients with SDANS, the basis of the pathogenesis of which is formed by the tension of the regulatory systems. Correlation between the total score according to the developed method, the RRNN value after 4 min of staying in orthostasis, and the “Myocardium” integral index has been proved. The diagnostic sensitivity of the proposed method with a threshold score of 8 was 80%, specificity — 70.8%.

**Conclusion.:**

The developed method for assessing pre-nosological changes in the electrophysiological state of the myocardium which includes cardiovascular risk factors with a reclassifying potential, proves the development of pre-nosological changes in patients with SDANS in response to daily physical strain. The changes are associated with the tension of the electrophysiological state of the myocardium, an increased activity of the sympathetic division of the ANS being one of its pathogenetic mechanisms.

## Introduction

The development of new methods for the diagnosis of cardiovascular diseases (CVD) continues to be an urgent problem [1, 2]. Currently, there is evidence of the impact of serious mental diseases, such as bipolar disorder and schizophrenia, on the risk of CVD [3]. In this regard, specialists in practical medicine pay more and more attention to borderline mental disorders: somatoform (psychosomatic) and neurotic ones, associated with stress. Behavioral and psychosocial risk factors are common in patients with such disorders, however they are more likely to be corrected than in patients with serious mental illnesses. This fact allows a greater efficiency of preventive interventions and, consequently, reduction of the risk of CVD in these individuals [4–6]. Therefore, we consider it important to study the probability of developing pre-nosological conditions and, possibly, an increased risk of CVD as well as to develop a method for their diagnosis in patients with somatoform dysfunction of the autonomic nervous system (SDANS) and CVD risk factors.

**The aim of the study** was to develop a method for diagnosing pre-nosological changes in the electrophysiological state of the myocardium (EPSM) using the ECG dispersion mapping method in patients with somatoform dysfunction of the autonomic nervous system and risk factors for cardiovascular diseases.

## Materials and Methods

The work was carried out at the Voronezh State Medical University named after N.N. Burdenko at the Department of Polyclinic Therapy and General Medical Practice. A total of 109 male patients were examined, including 58 subjects with SDANS aged 22.9±1.6 years (the main group) and 51 healthy men aged 22.8±2.0 years (the control group).

The diagnosis of SDANS was based on the criteria of neurocirculatory asthenia proposed by V.I. Makolkin and S.A. Abakumov (1996). The CVD risk factors were analyzed in conformity with the “Cardiovascular Prevention 2017” National Guidelines developed by the Committee of Experts of the Russian Cardiological Society and Russian National Society for Cardiovascular Prevention and Rehabilitation. The study of the functional reserves was based on the R.M. Baevsky’s classification (2003), according to which there are four main states of the regulatory systems: physiological norm, pre-nosological, premorbid states, and disruption of adaptation mechanisms with disease development.

EСG dispersion mapping was used as a method of pre-nosological diagnosis. The EPSM was studied by the values of the “Myocardium” integral index. The values of the integral index ≤14% at rest and ≤17% after the physical stress test were interpreted as normal. The values equal to 15–19% represented the borderline state associated with the EPSM tension (pre-nosological state). The values of the integral index of 20–25% and >25% showed a premorbid state associated with the EPSM overstrain and depletion of the reserves of the myocardial functioning with signs of the disease, respectively [7].

All the subjects involved in the study underwent two functional tests: a physical stress test and an active orthoclinostatic test (AOCT). The day before the functional tests, strong tea, coffee, and alcohol were excluded. The physical stress test consisted of the baseline examination within 60 s at rest and then 2 and 4 min after physical exertion respectively at examination points T1, T2, T3, and T4. The AOCT was performed according to the following algorithm: initial examination in a lying position (T5); in an upright position (T6); 2 min (T7) and 4 min (T8) after staying in orthostasis; in a horizontal position (T9); 2 min (T10) and 4 min (T11) after the transition to the horizontal position.

**The statistical data processing** was carried out using the Statistica software package for Windows and IBM SPSS Statistics 20.0. The p=0.05 value was taken as the threshold level of statistical significance. The Shapiro–Wilk test was used to verify the normality of the analyzed data and the equality of the variances of the feature distributions in the compared groups. In the case of the distribution of the studied features in accordance with the normal law, the mean value (M) was taken as the most typical indicator for the sample, and the standard deviation (σ) was taken as a measure of dispersion. The median and interquartile range were used as a measure of the central tendency and a measure of dispersion in the event that the studied features did not obey the normal law. The correlation between the quantitative normally distributed data was investigated with the Pearson’s method, the relationship between the quantitative abnormally distributed data was measured using Spearman’s correlation (R). Logistic regression was used for the theoretical verification of the developed method and determination of the threshold score. The ROC analysis was used to assess the predictive power of the method and analysis for sensitivity and specificity.

## Results

**Figure 1:**
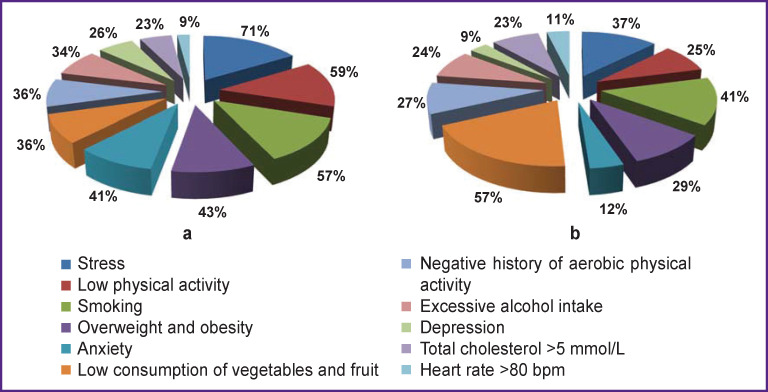
The structure of risk factors in men with cardiovascular diseases: (a) in patients with SDANS; (b) in healthy subjects

Among the CVD risk factors in men with SDANS, psychosocial stress, low physical activity, smoking, overweight and obesity were the leaders (Figure 1 (a)). In healthy men, the structure of risk factors was different: in 61% of cases, they noted a positive family history (in the first-degree relatives) of early manifestation of IHD or CVD. Insufficient consumption of vegetables and fruit (excluding potatoes), smoking, and psychosocial stress in healthy subjects were found in 57, 41, and 37% of cases, respectively (Figure 1 (b)).

The male patients with SDANS reported the following complaints: stabbing pain in the heart — 48 (82%); respiratory disorders manifesting themselves in a feeling of having a lack of air or lack of breath sensation — 46 (80%). Pulse and blood pressure lability occurring spontaneously or inadequately to physical activity, complaints of palpitation or pulsation in the precordial area or in the neck vessels, vegetative-vascular symptoms (redness, paleness of the face, cold extremities, marbling of the skin), low physical performance, increased fatigue and weakness occurred in 31 (54%), 27 (47%), 23 (39%), and 32 (55%) patients, respectively. The analysis of some clinical parameters in the patients with SDANS and healthy subjects, the “Myocardium” integral index values, the average duration of R–R intervals (RRNN) during the physical stress test and AOCT showed no statistically significant differences between the groups (Table 1).

**Table 1:** Some clinical features, values of the “Myocardium” integral index and RRNN in men with SDANS and healthy subjects

Clinical features	Patients with SDANS (n=58)	Healthy subjects (n=51)
HR per minute (M±σ)	76.5±10.0	72.0±10.1
SBP (mm Hg) (M±σ)	129.1±16.4	128.6±11.9
DBP (mm Hg) (M±σ)	88.3±10.1	88.6±8.8
Total cholesterol (mmol/L) (M±σ)	4.2±1.0	4.0±0.6
“Myocardium” integral index (p±σp%):		
Т1	13.8±5.4	13.8±2.6
Т2	16.0±6.2	15.6±4.8
Т4	15.7±6.2	16.0±6.7
Т8	17.3±9.8	15.6±5.6
Т11	13.2±5.3	13.5±3.0
RRNN:		
Т1	791.3±110.2	855.0±137.8
Т2	656.2±86.5	710.7±135.0
Т4	769.1±109.8	823.9±140.7
Т8	690.4±83.9	734.5±115.8
Т11	901.6±120.1	955.7±155.1

To develop a method for diagnosing pre-nosological changes in the EPSM in patients with SDANS, the severity of the studied CVD risk factors was ranged from 0 to 3 points. If the office heart rate in a patient with SDANS was 50–59 per minute, it was scored 0 and suggested high cardiovascular fitness of the vascular system. When the office heart rate fluctuated between 60–69, 70–79, or was ≥80 per minute, 1, 2, or 3 points were given, respectively. In this case, the fitness of the cardiovascular system in the subjects was determined as good, satisfactory, and unsatisfactory. 2 points were given and a high level of stress was suggested if the stress level according to the questionnaire by L.G. Reeder et al. (1969) was 1–2. If the stress level was 2.01–3.0 or 3.01–4.0, 1 or 0 points were given and medium and low stress levels were suggested, respectively. The total response on the anxiety subscale (HADS scale) of 0–5, 6–9, ≥10 was given 0, 1, or 2 points, respectively, which characterized the absence of anxiety, subclinical or clinically pronounced anxiety. On the HADS depression subscale, the total response of 0–5, 6–9, ≥10 was interpreted as 0, 1, and 2 points, respectively, suggesting no depression, subclinical or clinically significant depression. If a patient with SDANS smoked ≥1 cigarette per day, 2 points were given, <1 cigarette per day — 1, with a negative smoking status — 0 points.

The physical activity level according to R.A. Potemkina (2012) was determined by a positive answer to questions 1–4, 5–6, and 7–8, with the assignment of 2, 1, and 0 points, respectively. If the patient answered “yes” to questions 1–4, it suggested low activity, questions 5–6 — moderate activity, 7–8 — intense physical activity. The values of body mass index (BMI) of 18.5–24.9, 25.0–29.9, and ≥30, respectively, were assigned 0, 1, and 2 points: 0 points suggested normal BMI, 1 — overweight, 2 — obesity.

If the office BP was ≤129/84 mm Hg, it was given 0 points, if the SBP was 130–139 mm Hg and DBP was 85–89 mm Hg, it was given 1 point, interpreted as normal and high blood pressure, respectively. If SBP and DBP ≥140/90 mm Hg, it was given 2 points.

The daily alcohol consumption of <2 servings in the patients with SDANS was assigned 0 points, 2 and >2 servings were evaluated as 1 and 2 points, respectively.

We studied the past history of extra aerobic physical activity during childhood and/or adolescence. If the history was positive, it was given 0 points, it being negative — 1 point.

Negative and positive family history of IHD or CVD in the first-degree relatives (men <55 years old and women <65 years old) was interpreted as 0 and 1 points, respectively.

If the subject ate ≥5 servings of vegetables and fruit per day, excluding potatoes, 0 points were given, if <5 servings, 1 point was given (Table 2).

**Table 2:** Pre-nosological changes in the electrophysiological state of the myocardium in patients with SDANS detected with the proposed method

Risk factor	Risk factor severity (points)		
	0	1	2
Heart rate at rest, per minute (if heart rate at rest ≥80, scored 3)	50–59	60–69	70–79
Depression level (HADS)	0–5	6–9	≥10
Anxiety level (HADS)	0–5	6–9	≥10
Office ABP (mm Hg)	≤129/84	130–139/85–89	≥140/90
Smoking status (cigarettes/day)	Negative	<1	≥1
Severity of stress (by L.G. Reeder et al.)	3.1–4.0	2.1–3.0	1.0–2.0
Level of physical performance (by R.A. Potemkina)	Answers 7–8	Answers 5–6	Answers 1–4
Alcohol consumption (standard dose/day)	<2	2	>2
Body mass index	18.5–24.9	25.0–29.9	≥30.0
History of extra aerobic physical activity in childhood and/or adolescence	Positive	Negative	—
Consumption of vegetables and fruit per day (excluding potatoes)	≥5 servings	<5 servings	—

The value of RRNN after 4 min of orthostasis (T8) and the value of the “Myocardium” integral index after physical exertion (T2) were selected as the reference points in the development of the method which was based on the literature data [5, 7]. According to it, RRNN is the reciprocal of heart rate, and it is this indicator that shows an increase in heart rate due to the activation of the sympathetic division of the ANS and the release of catecholamines into the blood after 4 min in orthostasis (T8). The “Myocardium” integral index after physical exertion (PE) (T2) allows to assess the nature of the response of the cardiovascular system to daily PE and detect transient changes in this system in patients with SDANS without clinical manifestations of CVD.

We had to prove the statistical significance of the correlation between the total score for all the CVD risk factors and the RRNN value after 4 min in orthostasis (T8), between RRNN value in T8 and the “Myocardium” integral index after PE (T2) and, finally, between the “Myocardium” integral index in T2 and the total score for CVD risk factors. As a result, according to our method, we established a statistically significant inverse moderate (r=–0.41; p<0.05) correlation between the total score for all CVD risk factors and RRNN in T8. This proves that the greater the total score, the lower the RRNN value in T8, i.e. the higher the activity of the sympathetic part of the ANS, is. Having obtained a moderate correlation between RRNN in T8 and the “Myocardium” integral index in T2 (r=–0.25; p<0.05), we demonstrated that the higher the activity of the sympathetic part of the ANS, the greater the value of the “Myocardium” integral index after PE (T2) is. And, finally, our method proves the presence of a direct moderate correlation between the total score for CVD risk factors and the “Myocardium” integral index in T2 (r=0.54; p<0.05) in the patients with SDANS. This, in its turn, proved that the higher the total score, the higher the value of the “Myocardium” integral index after PE is.

Thus, the total score for CVD risk factors of more than 8 in men suffering from SDANS corresponds to the “Myocardium” integral index value after PE of ≥18% and shows the tension of the regulatory systems with the development of pre-nosological conditions.

The theoretical verification of the method was carried out using binary logistic regression. The dependent variable in the patients with SDANS was the presence of pre-nosological changes in the EPSM, and the only predictor was the total score calculated with the use of the developed and studied method (see Table 2).

The regression equation looks as follows:


$$\rho =\frac{1}{1+e^{8.000-0.767x}},$$

where ρ is the theoretical probability of pre-nosological changes in the EPSM in patients with SDANS according to the developed method; *x* is the value of the total score for CVD risk factors in a patient with SDANS.

This regression model predicts the risk of developing pre-nosological changes in the EPSM in patients with SDANS with an accuracy of 89.7%. The Cox and Snell’s R^2^ coefficient of determination is 0.269; Nagelkerke’s R^2^ is 0.447; –2Log likelihood ratio is 35.178; χ^2^=18.146; p<0.001; the level of statistical significance of the regression coefficients is p<0.05.

The prediction accuracy in the patients with SDANS with independent inclusion of predictors in the regression model was the same as in the model based on the total score and amounted to 89.7%. However, the coefficients of determination were slightly greater: Cox and Snell’s R^2^ was 0.330; Nagelkerke’s R^2^ was 0.549; –2Log likelihood ratio was 30.109; χ^2^=23.216; p<0.05; the level of statistical significance of the regression coefficients was p>0.05.

Therefore, the exact model was determined by the total score according to our method, serving the only integral categorized predictor. The theoretical probability of the presence of pre-nosological changes in the EPSM for each patient was calculated using the above regression equation.

**Figure 2:**
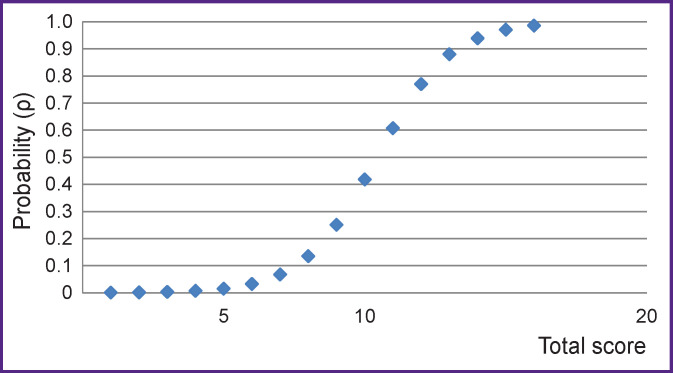
Dispersion diagram. Dependence of the theoretical probability of the presence of pre-nosological changes in the electrophysiological state of the myocardium in patients with SDANS with risk factors for cardiovascular diseases on the total score according to the developed method

In the same way, we calculated the mean probability values for each patient with SDANS and determined the range of theoretical probability values at which the pre-nosological changes in the EPSM were practically not detected in the sample. The range of the theoretical probability values from 0.0061 to 0.2095 characterized the interval at which pre-nosological changes in EPSM in the sample were not practically detected. On the graph (Figure 2), it was determined that the range of the theoretical probabilities at which pre-nosological changes in EPSM in the sample did not practically occur corresponds to the interval from 0 to 9 points, and the average value of this probability (0.1078) falls within the interval from 8 to 9 points.

**Figure 3:**
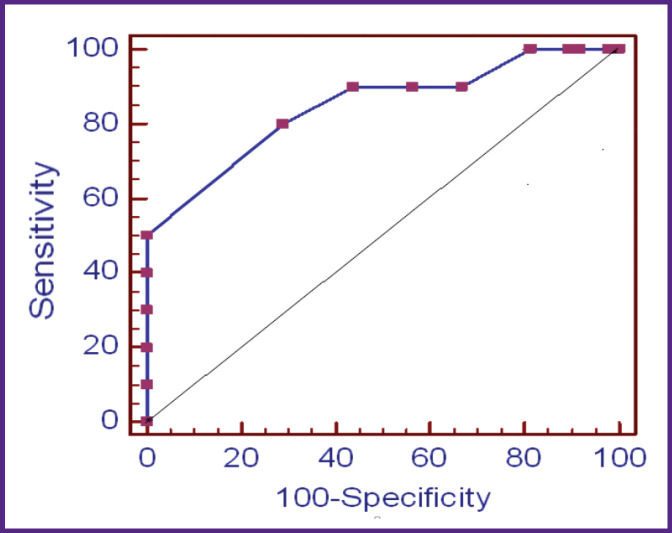
ROC-curve of the method for diagnosing pre-nosological changes in the electrophysiological state of the myocardium in patients with SDANS with risk factors for cardiovascular diseases

**Figure 4:**
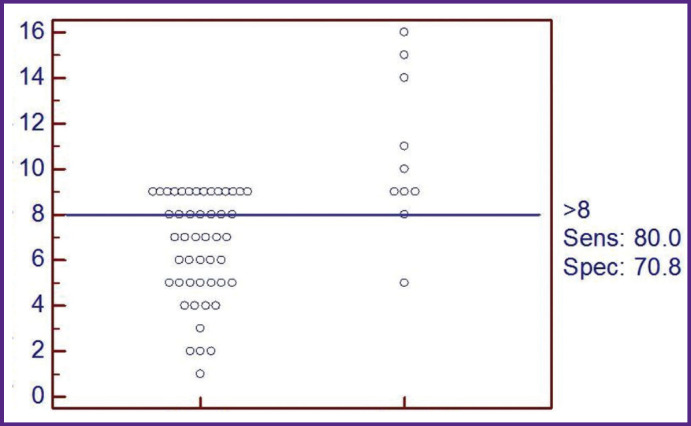
Optimal cut-off thresholds depending on patient distribution by the risk level and the value of the total score for the method of diagnosing preclinical changes in the electrophysiological state of the myocardium in patients with SDANS

Further, the threshold score, its diagnostic sensitivity, and specificity were determined by the ROC analysis method for the patients with SDANS. Using the ROC analysis of the sensitivity and specificity indicators and plotting the characteristic curve (Figure 3), we revealed a good predictive ability of this model for calculating the probability of pre-nosological changes in EPSM in male patients with SDANS. The curve indicated the area of 0.846±0.076 (95% CI 0.696–0.996; p<0.0001). The diagnostic sensitivity was 80%, the specificity was 70.8% with a threshold score of 8 (Figure 4).

## Discussion

Throughout the entire study of SDANS, the researchers were interested in the relationship between mental and somatic disorders, and the CVS symptoms occurring in the patients were studied most closely. In our study, using ECG dispersion mapping as a new method of preclinical diagnosis, we studied the pathogenesis of SDANS in male patients with CVD risk factors. It was established that having the total score of more than 8 for CVD risk factors according to this method, patients with SDANS develop pre-nosological conditions in response to daily physical strain associated with the tension of the EPSM. One of the pathogenetic mechanisms of pre-nosological conditions is an increase in the activity of the sympathetic division of the ANS [4, 6]. Consequently, psychosocial risk factors for CVD in combination with behavioral and biological ones, through an increase in the activity of the sympathetic division of the ANS and an increase in heart rate, lead to hemodynamic and neurohumoral shifts in the functioning of the cardiovascular system. In the absence of timely correction of CVD risk factors, the adaptive reserves of the “concerned” organ, the cardiovascular system in our study, are gradually depleted. In this case, the general adaptation syndrome with the development of CVD enters the pathological stage.

The limited set of risk factors is a drawback of the modern scales used to assess cardiovascular risks [1, 2, 8]. Our method takes into account risk factors with a reclassifying potential. According to the latest Guidelines for Cardiovascular Prevention (2017), the presence of risk factors with a reclassifying potential in patients with SDANS can increase or decrease cardiovascular risk [9]. With our method, a patent was obtained for the invention of the “Method for evaluating high risk of cardiovascular diseases in young individuals” [10]. We are planning to test the model in the clinic.

## Conclusion

Thus, a patient with somatoform dysfunction of the autonomic nervous system continues to be a difficult patient for the clinician. The interdisciplinary approach is needed in the management of these patients with close interaction between the primary care physician who is most often seen by the patient, the cardiologist, and the mental health professional who must interact on the basis of the biopsychosocial model of the integration of somatic, therapeutic factors and psychosocial determinants.

The method developed on the basis of ECG dispersion mapping will improve the efficiency of preventive interventions in patients with borderline mental disorders.
